# Angiolytic laser stripping versus CO2 laser microflap excision for vocal fold leukoplakia: Long-term disease control and voice outcomes

**DOI:** 10.1371/journal.pone.0209691

**Published:** 2018-12-31

**Authors:** Jae-Yol Lim, Young Min Park, Minsuk Kang, Seung Jin Lee, Kwangha Baek, Jina Na, Hong-Shik Choi

**Affiliations:** Department of Otorhinolaryngology, Gangnam Severance Hospital, Yonsei University College of Medicine, Seoul, Republic of Korea, Republic of Korea; Seoul National University Hospital, REPUBLIC OF KOREA

## Abstract

**Background and purpose:**

Vocal fold leukoplakia, white plaque on the epithelium, has the potential for malignant transformation regardless of dysplasia grade. It is treated with different laser types (CO_2_ or angiolytic) and various techniques (vaporization, stripping, or excision); however, only a few studies exist regarding comparative laser surgery results. This study was conducted to investigate clinical outcomes of CO_2_ versus angiolytic laser microdissection with regard to long-term disease control and voice preservation in vocal fold leukoplakia.

**Materials and methods:**

Seventy patients with vocal fold leukoplakia treated by CO_2_ or angiolytic laser (pulsed dye laser or potassium titanyl phosphate) were identified retrospectively. Data regarding patient characteristics, treatment details, treatment outcomes including disease control (recurrence and progression) and the Voice Handicap Index, GRBAS scale, and acoustics were evaluated. The mean follow-up duration after initial treatment was 32 ± 26 months.

**Results:**

The study group comprised 14 patients who underwent CO_2_ laser microflap excision and 56 who underwent angiolytic laser stripping. Of the patients treated with CO_2_ laser, 11 (79%) had no recurrence and three (21%) showed recurrent leukoplakia, of which one patient (7%) showed histologic grade progression. Of patients who underwent angiolytic laser stripping, 12 had disease recurrence (21%), among whom three (5%) showed disease progression. Laser surgery type, disease extent, and histologic grade showed no significant differences in recurrence or progression rates. The postoperative Voice Handicap Index significantly improved (*P* = .03) and the G score significantly decreased (*P* < .001) in the angiolytic laser treatment group. In contrast, the Voice Handicap Index increased postoperatively in the CO_2_ laser group (*P* = .046).

**Conclusions:**

The long-term recurrence or progression rates were not significantly different between angiolytic and CO_2_ laser treatment. The angiolytic laser stripping group showed better voice preservation compared with the CO_2_ laser group. Angiolytic laser stripping is suggested as an effective treatment option for vocal fold leukoplakia with comparable disease control and better voice preservation.

## Introduction

Vocal fold leukoplakia is clinical terminology indicating whitish plaques on the surfaces of the vocal folds. Vocal fold leukoplakia encompasses a variety of pathologic changes such as (hyper)keratosis, dysplasia, carcinoma in situ (CIS), and cancer. Keratosis is an abnormal pathologic change resulting from the production of keratin on the vocal fold epithelium. Dysplasia refers to abnormal keratinization on the epithelium of the vocal folds with cellular and architectural atypia; this is considered a premalignant lesion that may lead to CIS and invasive squamous cell carcinoma. The grade of dysplasia is assessed as mild, moderate, or severe based on the thickness of the epithelium with cellular and architectural atypia.

A systematic review of studies from 1960 to 2005 on 2,188 patients with laryngeal leukoplakia showed that approximately 50% of laryngeal leukoplakia cases were benign and 50% of cases were premalignant or malignant lesions [1]. Overall, 8.2% underwent malignant transformation and the grade of dysplasia correlated with the rate of malignant transformation: 3.8% in no dysplasia, 10.1% in mild or moderate dysplasia, and 18.1% in severe dysplasia or CIS. A recent meta-analysis of 940 cases of laryngeal dysplasia also revealed that the overall malignant transformation rate became higher according to increasingly severe dysplasia grades; that of mild or moderate dysplasia was 10.6% while that of severe dysplasia or CIS was 30.4% [2]. A single-institution study at Johns Hopkins Hospital revealed that the incidence of mild dysplasia increased and that of CIS decreased in a more recent cohort, while the rate of progression of dysplasia to cancer was unchanged (8.8% in a previous 10-year period and 8.0% in the most recent 10-year period) [3].

Although vocal fold leukoplakia possesses malignant potential, determining the surgical extent remains challenging due to the high incidence of benign keratosis in vocal fold leukoplakia and the relatively low rate of malignant transformation of premalignant vocal fold dysplasia. Moreover, epithelial scarring and changes in components of the layered structures of the vocal folds result in mucosal vibration disturbance and lead to irreversible dysphonia. Considering these clinical dilemmas, microdissection of whitish plaques on vocal folds using lasers or cold instruments has been considered an appropriate surgical treatment for vocal fold leukoplakia. Recently, angiolytic lasers have been employed in vocal fold leukoplakia and the surgical outcomes have shown favorable results [4–8]. However, there is little literature regarding the efficacy of angiolytic laser treatment with regard to therapeutic outcomes, especially in comparison with CO_2_ laser microflap excision, which is the most commonly used surgical tool in the treatment of vocal fold leukoplakia.

To report our experience and outline a treatment plan for vocal fold leukoplakia, we determined the clinical efficacy of laser microdissection with CO_2_ versus angiolytic lasers. The primary outcome measure was long-term disease control (recurrence or progression) and the secondary outcome measure was voice preservation after vocal fold leukoplakia treatment. Our results will provide important clues for refining surgical techniques in the treatment of vocal fold leukoplakia.

## Methods

The Institutional Review Board of Yonsei University approved our retrospective study and waived the requirement for patient informed consent. Patients who were treated with lasers for vocal fold leukoplakia between 2006 and 2016 were identified from our database and their medical records were reviewed retrospectively. Pathologic reports on biopsies from surgical specimens were reviewed, and patients who were finally diagnosed with a benign (keratosis without dysplasia) or premalignant lesion with the presence of dysplasia were enrolled. Cases of invasive squamous cell carcinoma or pathologic reports without confirmation because of insufficient material or artifacts were excluded. We only included patients treated by laser microdissection; cases in which the surgical extent exceeded a type II cordectomy and cases that included combined modalities with cold instruments were excluded. Patients referred for recurrent leukoplakia who underwent surgery previously were also excluded.

Demographic information (age, sex, and smoking history), human papillomavirus (HPV) infection, disease extent (unilateral or bilateral), laser treatment type and settings, follow-up duration, and final disease state were reviewed from medical records. Patients were grouped into the CO_2_ and angiolytic laser groups. CO_2_ laser surgery was conducted during the earlier period of this study; the surgical modality transitioned to angiolytic laser surgery after pulsed dye laser (PDL) and potassium titanyl phosphate (KTP) were introduced at our institute.

**Fig 1:**
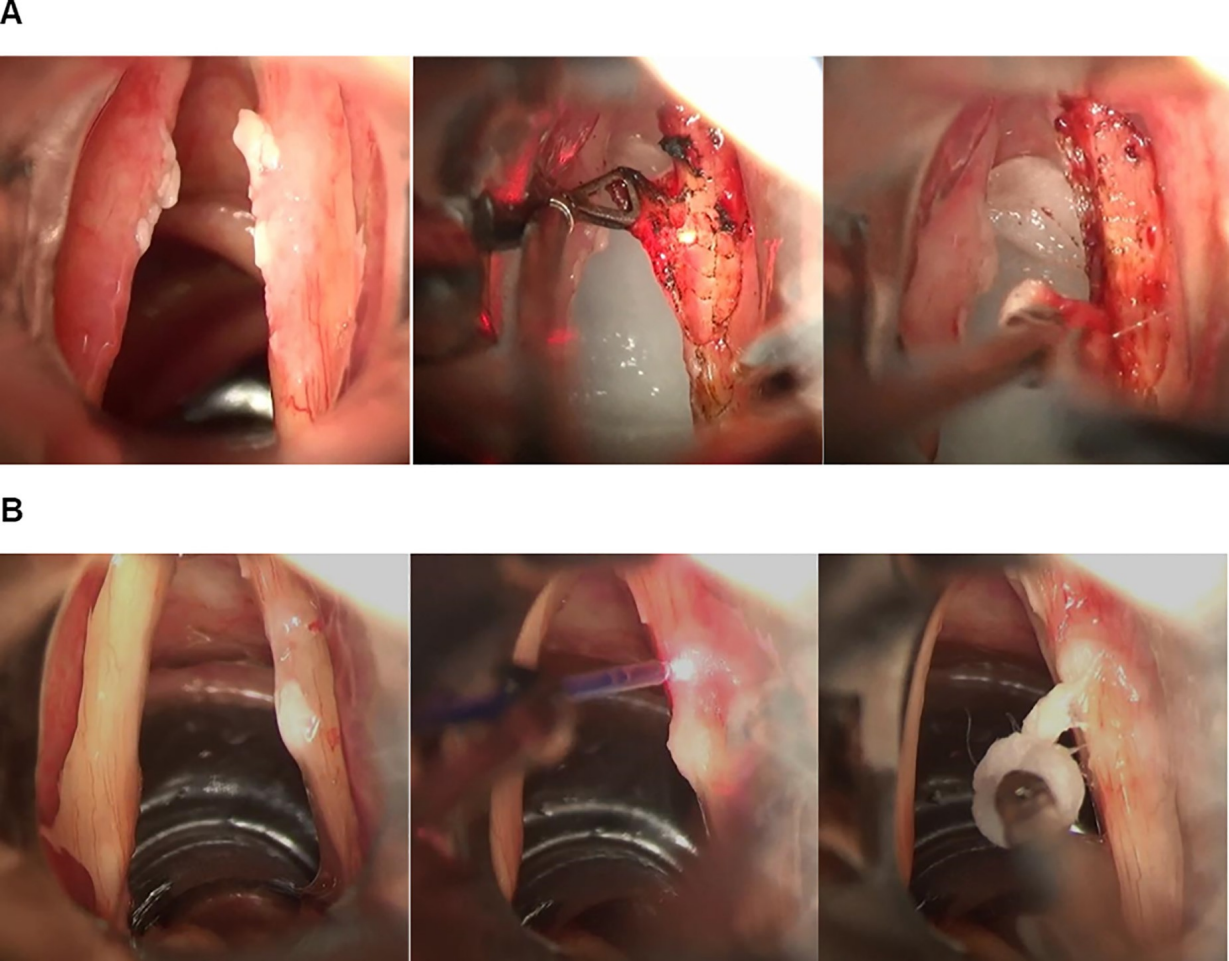
The surgical processes of (A) CO_2_ microflap excision and (B) angiolytic stripping for vocal fold leukoplakia.

We conducted laser microdissection as follows: a CO_2_ microflap excision was performed under general anesthesia. The true vocal folds were exposed using a laryngoscope and a magnified view of the operative field was obtained by using a surgical microscope. After evaluation of the disease extent based on the magnified view, the leukoplakic lesion on the mucosa was circumferentially incised around the margin of the lesion using a CO_2_ laser (Sharplan 1041, GA, USA) in 4-W super-pulse mode. A microflap was made along the subepithelial plane such as in a type I cordectomy and the entire lesion was removed using microsurgical instruments (Fig 1A).

Angiolytic irradiation and stripping were performed as described previously [6]. In brief, after evaluation of the magnified vocal fold leukoplakia lesions under general anesthesia, a flexible laser fiber was inserted for transmission of the angiolytic laser. The tip of the fiber was grasped using alligator forceps and moved to its target within 3–5 mm of the lesion. An angiolytic laser (PDL or KTP) was used to deliver 50–70 pulses with a mean output of 600 (range, 100–700) mJ per pulse. After irradiating the entire lesion, a cleavage plane was created between the basement membrane and leukoplakic epithelium, which was separated from the underlying superficial lamina propria by denaturing the basement membrane zone linking proteins. Then, the leukoplakic lesion can be readily elevated and stripped from the vocal folds using a small cotton ball and grasping forceps (Fig 1B). Additional biopsies were performed at the margins using micro scissors and the specimens were sent to the department of pathology for histopathologic evaluation and HPV DNA testing.

Cases were divided by disease extent into the unilateral and bilateral leukoplakia groups. Pathologic grades were rated as no dysplasia, mild/moderate dysplasia, or severe dysplasia/CIS. The disease control rate was evaluated according to correlations with the treatment modality (CO_2_ laser versus angiolytic laser), disease extent (unilateral versus bilateral), and histologic grade (no dysplasia vs. dysplasia or mild/moderate vs. severe/CIS). During follow-up, patients with disease disappearance or regression were defined as responders and those with recurrence or with persistent or progressing lesions were defined as non-responders. Non-responders with progression of pathologic grades were treated with serial laser surgeries with or without radiotherapy.

Voice assessments were conducted before and 3 months after treatment; they included auditory-perceptual evaluation, psychometric assessment, and acoustic analysis. For perceptual judgment, participants were asked to read a standard Korean passage called “autumn” aloud, which is approximately 2 minutes in length. The auditory-perceptual evaluation was conducted on the recorded samples using the Computerized Speech Lab (Model 4150B; KayPENTAX, NJ, USA) and GRBAS (grade, roughness, breathiness, asthenia, and strain) scale in a blinded manner by two certified speech-language pathologists who determined a score in consensus from 0 to 3 points (0, normal; 1, mild; 2, moderate; 3, severe). The Korean–Voice Handicap Index (K–VHI) was also administered to evaluate self-perceived vocal complaints from 0 to 4; higher scores indicate greater vocal handicaps. Acoustic analysis measurement was performed using the Multi-dimensional Voice Program (Model 5105, KayPENTAX). As acoustic parameters, the jitter percentage, shimmer percentage, and noise-to-harmonic ratio (NHR) were measured from a 4-second stable portion of /a/ vowel phonation samples.

The Mann–Whitney U test was used to compare continuous variables between the CO_2_ laser microflap and angiolytic stripping groups. Pearson’s chi-squared test or Fisher’s exact test was used to compare categorical variables. The Wilcoxon signed-rank test was used when comparing two matched samples in a single patient to assess whether the mean population ranks differed. The recurrence or progression-free rate was calculated by the Kaplan-Meier method. *P* < .05 was considered statistically significant. Statistical analyses were performed using the Statistical Package for the Social Sciences (SPSS) version 18.0 (SPSS, Inc., Chicago, IL, USA).

## Results

The mean age of all patients was 56.87 years; 68 patients were male and two patients were female. An angiolytic laser was used for stripping leukoplakia lesions in 56 patients and a CO_2_ laser microflap procedure was used in 14 patients. There were no statistically significant differences in age, sex, smoking history, HPV status, or pathologic findings between the CO_2_ laser microflap and angiolytic laser stripping groups (Table 1).

**Table 1:** Clinical information and treatment outcomes of all patients.

	**Angiolytic laser (n = 56)**	**CO**_**2**_ **laser (n = 14)**	**Total** ^**a**^	***P*-value**
**Age (years)**	56.05 ± 12.13	60.14 ± 12.94	56.87 ± 12.31	.26
**Sex**				.28
**Male**	55	13	68	
**Female**	1	1	2	
**Smoking (pack-years)**	19.77 ± 21.27	12.86 ± 18.68	18.39 ± 20.84	.27
**HPV status**				
**Positive**	2	0	2	.54
**Negative**	16	3	19	
**Not performed**	38	11	49	
**Pathology**				.13
**No dysplasia**	36	5	41	
**Mild dysplasia**	15	4	19	
**Moderate dysplasia**	2	2	4	
**Severe dysplasia/CIS**	3	3	6	

^a^ Includes 70 patients who underwent 56 anigiolytic laser stripping and 14 CO_2_ microflap excision.

**Fig 2:**
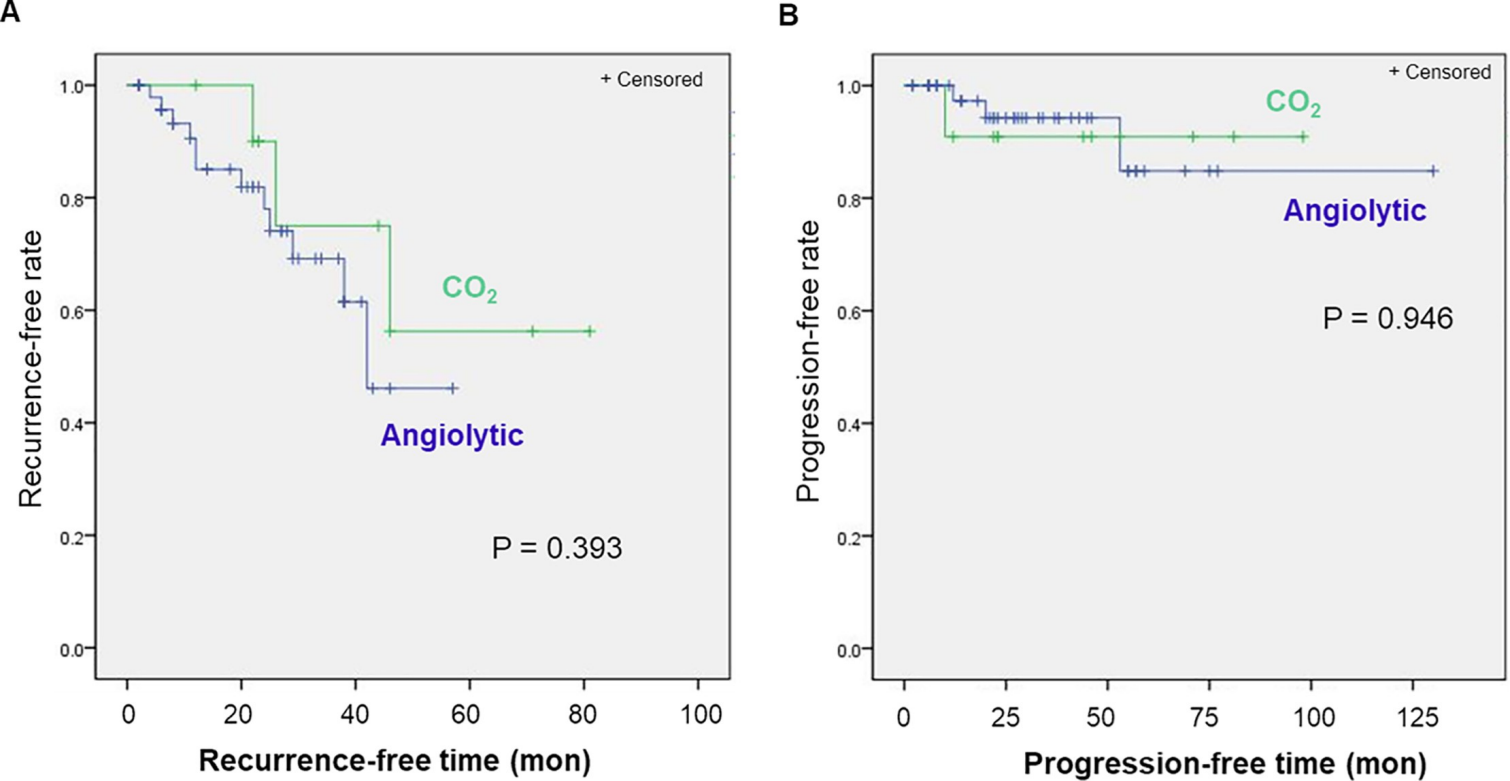
Kaplan-Meier Curve of (A) recurrence-free and (B) progression-free rate.

Patients were divided into two groups (responders vs. non-responders) according to disease control (Table 2). During the study period, 55 patients responded to the treatment in which the lesion disappeared or regressed during follow-up and 15 patients had recurrent, persistent, or progressive disease after the operation. Of the 14 patients treated using a CO_2_ laser, 11 patients (79%) had no recurrence and three patients (21%) showed recurrent leukoplakia. Of the 56 patients who underwent angiolytic laser stripping, 12 patients (21%) had disease recurrence. There was no significant difference in the ratio of responders to non-responders according to sex, smoking history, HPV infection status, treatment modality, disease extent, or dysplasia grade (Table 2). There was no significant difference in the recurrence-free curve between the angiolytic laser and the CO2 laser groups, with 40.99%, and 60.28% recurrence-free rate at 5 years in the angiolytic laser and CO_2_ laser group, respectively (Fig 2A).

**Table 2:** Clinicopathologic factors related to treatment response^a^.

	**Responders** **(n = 55)**	**Non-responders** **(n = 15)**	***P*-value**
**Sex**	Male: 53 Female: 2	Male: 15 Female: 0	.45
**Smoking** **(pack-years)**	17.77 ± 20.35	20.67 ± 23.13	.60
**HPV status^b^**	Positive : 2 Negative : 13 Not performed : 40	Positive : 0 Negative : 6 Not performed : 9	-
**Treatment modality**	Angiolytic: 44 CO_2_: 11	Angiolytic: 12 CO_2_: 3	>.99
**Disease extent**	Unilateral: 30 Bilateral: 25	Unilateral: 10 Bilateral: 5	.40
**Dysplasia grade**	No dysplasia: 34 Mild/moderate: 15 Severe/CIS: 6	No dysplasia: 8 Mild/moderate: 6 Severe/CIS: 1	.61

^a^ Includes 70 patients who underwent 56 angiolytic laser stripping surgeries and 14 CO_2_ microflap excision surgeries.

^b^ Statistical analysis was not performed because of insufficient data. HPV: Human papillomavirus.

The overall disease progression rate of vocal fold leukoplakia was 6% in our study; however, no lesion showed malignant transformation. Four of 15 patients with recurrence showed disease progression during the study period. Among the three patients with recurrence in the CO_2_ laser microflap group, one patient (33%) showed histologic grade progression. Among 12 patients with recurrence in the angiolytic laser group, three patients (25%) showed disease progression. There was no significant relationship between disease progression and any clinicopathologic factor such as sex, smoking history, HPV infection status, treatment modality, disease extent, or dysplasia grade (Table 3) The progression rate per year was not significantly different between the angiolytic laser and the CO_2_ laser groups (Fig 2B).

**Table 3:** Analysis of clinicopathologic findings related to malignant transformation^a^.

	No progression (n = 66)	Disease progression (n = 4)	*P*-value
**Sex**	Male: 64 Female: 2	Male: 4 Female: 0	.72
**Smoking** **(pack-years)**	15.00 ± 20.85	36.25 ± 24.62	.12
**HPV status^b^**	Positive : 2 Negative : 18 Not performed : 46	Positive : 0 Negative : 1 Not performed : 3	-
**Treatment modality**	Angiolytic: 53 CO_2_: 13	Angiolytic: 3 CO_2_: 1	.79
**Disease extent**	Unilateral: 37 Bilateral: 29	Unilateral: 3 Bilateral: 1	.46
**Dysplasia grade**	No dysplasia: 40 Mild/moderate: 19 Severe/CIS: 7	No dysplasia: 2 Mild/moderate: 2 Severe/CIS: 0	.59

^a^ Includes 70 patients who underwent 56 angiolytic laser stripping and 14 CO_2_ microflap excision.

^b^ Statistical analysis was not performed due to insufficient data.

**Fig 3:**
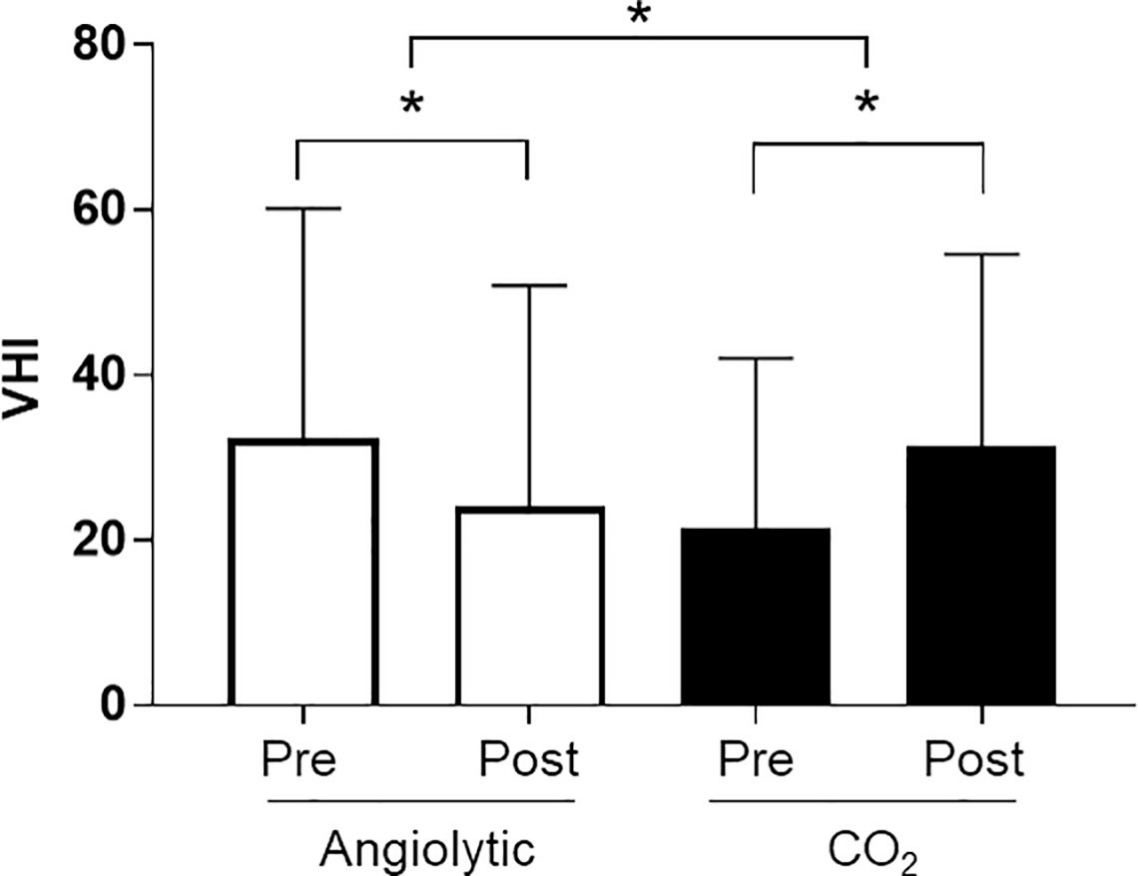
Voice Handicap Index scores before and after the operation.

**Fig 4:**
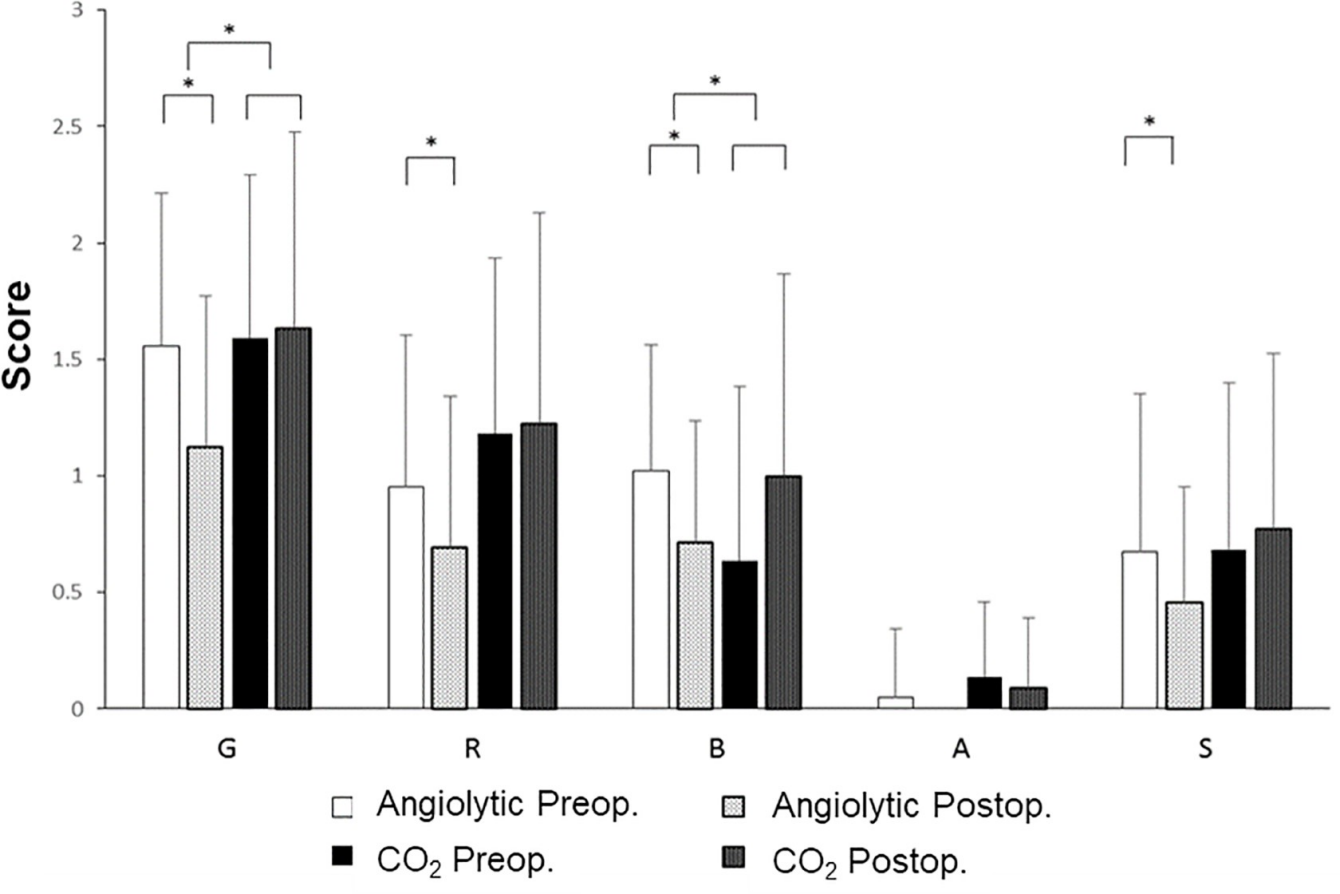
Preoperative and postoperative GRBAS (grade, roughness, breathiness, asthenia, and strain) scores.

Changes in the VHI before and after treatment were analyzed. In the angiolytic laser stripping group, there was a significant decrease in the VHI after treatment compared to the preoperative VHI value. However, in the CO_2_ laser microflap group, a significant increase in the VHI was observed after treatment compared with preoperative values (Fig 3). Changes in the GRBAS score between before and after treatment were also compared and analyzed. In the angiolytic laser group, the G, R, B, and S scores were decreased after treatment compared to before treatment, but no significant difference was observed in the CO_2_ laser group between before and after the operation (Fig 4). In the analysis of acoustic parameters measured using the Multidimensional Voice Program, no significant differences were found between the angiolytic laser group and the CO_2_ laser group (Table 4).

**Table 4:** Acoustic parameters measured by Multi-dimensional Voice Program before and after the operation.

		Preoperative	Postoperative	Mean of the differences	*P*-value
**Noise-to-harmonic ratio**	Angiolytic	0.167	0.181	-0.014	.82
	CO_2_	0.175	0.174	-0.001	
**Jitter (%)**	Angiolytic	2.54	2.36	0.18	.067
	CO_2_	2.09	3.69	-1.60	
**Shimmer (%)**	Angiolytic	5.47	5.46	0.01	.36
	CO_2_	5.60	7.23	-1.63	

## Discussion

Laryngeal dysplasia may progress to malignancy, although a relatively small proportion of dysplasia cases are eventually judged as premalignant lesions. Thus, management of vocal fold leukoplakia should encompass removal of whitish plaques from the vocal fold epithelium and biopsy once infectious causes such as fungal laryngitis are ruled out. Surgical modalities include ablation, stripping, and excision according to the extent of the surgical specimen removal. A CO_2_ or angiolytic laser can be applied to these various surgical techniques for the treatment of vocal fold leukoplakia. However, there is no consensus on the management of vocal fold leukoplakia because of the clinical dilemma of balancing oncologic safety with voice preservation after treatment. Furthermore, although there may be a temporal trend toward the emergence of microflap excision and/or photoangiolysis, the choice of surgical option depends on the surgeon’s preference and experience as well as the equipment and instruments available at the hospital [3].

Along with recent advances in laser surgeries, photoangiolytic surgeries using a PDL or KTP laser have been applied to various vocal fold lesions. These lasers can be used for ablation of the intra- and sublesional microcirculation without excision [5, 8]. Angiolytic lasers feature selective photothermolysis of aberrant and/or abundant microvasculature without altering the layered structure because the wavelengths of 585 nm and 532 nm used in PDL and KTP lasers, respectively, are near the absorption peak of hemoglobulin [5, 7]. We recently introduced a stripping technique for vocal fold leukoplakia using a PDL angiolytic laser [6]. We first irradiate the leukoplakic lesion for selective coagulation of the subepithelial microvasculature as well as separation of the epithelium from the underlying superficial lamina propria by denaturing the basement membrane zone linking proteins. Then, we elevate and strip the whitish plaque off using cotton balls and grasping forceps. In contrast, a CO_2_ laser vaporizes the water in tissues, thus enabling the cutting and excision of lesions. Traditionally, a CO_2_ laser has been used for cordectomy in early glottic cancer. Although it may induce more thermal damage to the normal tissue of the vocal fold lesions relative to angiolytic lasers, a previous study reported that it allows subepithelial microflap dissection such as in a type I cordectomy with a favorable vocal outcome [9].

In this study, we investigated the efficacy of laser microdissection (CO_2_ microflap excision vs. PDL or KTP angiolytic stripping) with regard to disease recurrence and progression. Our results showed that the laser treatment modality did not affect disease recurrence rates; 12 of 56 patients (21%) in the angiolytic group and three of 14 patients (21%) in the CO_2_ laser group had disease recurrence. The disease progression rate in the angiolytic laser stripping group (three of 56 patients, 5%) was comparable to that in the CO_2_ laser microflap group (one of 14 patients, 7%). These favorable results suggest that angiolytic laser stripping was an effective and safe treatment for vocal fold leukoplakia. In a retrospective study of 63 patients undergoing submucosal resection using cold microlaryngeal instruments or CO_2_ lasers, Lee et al. demonstrated that the degree of dysplasia and the extent of the lesion were factors predictive of recurrence [10]. A recent meta-analysis indicated that treatment modality did not show a significant effect on malignant transformation, but the risk of malignant transformation was higher with increasingly severe dysplasia grades; the mean time to transformation was 5.8 years [2]. On the contrary, no predictive factor related to disease severity affected disease recurrence or progression in this study. Further investigation is still required to interpret these results because this study was performed retrospectively with a small number of patients.

In the subjective voice evaluation, the angiolytic laser stripping group showed a significant improvement in subjective voice status compared with that in the CO_2_ laser microflap group. Moreover, CO_2_ laser surgeries showed worse voice outcomes according to the VHI and perceptual grade. A significant improvement in pitch perturbation quotient among acoustic parameters was recently described after KTP laser therapy for laryngeal dysplasia [7]. However, in the present study, acoustic values (jitter, shimmer, and NHR) did not differ significantly between the two treatment modalities, although antiolytic laser did not significantly impair acoustic parameters. Given that thermal damage to the normal tissues of the vocal folds is inevitable when using the CO_2_ laser microflap technique, voice quality can become more deteriorated postoperatively after CO_2_ laser microflap surgery than after angiolytic laser stripping surgery. However, additional investigation is required to verify the potential photothermal injury by angiolytic laser to delicate extravascular vocal fold tissues, according to the power setting of laser energy delivered and exposure time.

Angiolytic laser surgeries have been conducted for the treatment of various vocal fold lesions such as hemorrhagic polyps, nodules or cysts, and scars or sulcus [11–13]. Furthermore, office-based surgeries under local anesthesia have been advocated for the treatment of laryngeal dysplasia or papilloma [5, 14–17]. Office-based laser irradiation under local anesthesia may be less efficacious as a definitive treatment modality than that under general anesthesia because of difficulty in positioning the laser fibers and quantifying the energy as well as the inability to obtain biopsy samples for histologic analysis. Nevertheless, an office-based setting following initial treatment in the operating room and obtaining a biopsy specimen appears to facilitate proper disease control with minimal morbidity [3, 8].

This study includes some limitations due to its retrospective study design as follows.

The small number of patients in CO_2_ laser surgery may preclude a definite conclusion regarding factors predictive of disease recurrence and progression. Disease control was determined by subjective laryngoscopic findings of leukoplakic lesions during follow-up, although disease persistence or progression was evaluated relative to the initial endoscopic findings and pathologic reports. It was also difficult to conclude that angiolytic laser stripping outweigh CO_2_ laser microflap surgery in regards to quantitative voice quality, possibly due to the relatively small number of patients who underwent CO_2_ laser microflap surgery. In addition, selection bias may be present because surgeons may have chosen treatment with a CO_2_ laser for relatively more extensive lesions during an earlier period when there was overlap in the use of CO_2_ and angiolytic lasers. Nevertheless, this study highlights for the first time that long-term disease control using angiolytic laser stripping for the treatment of vocal fold leukoplakia was favorable and postoperative subjective voice outcomes were better than those in CO_2_ laser microflap surgery.

In our study, the angiolytic laser group showed not only comparable results in terms of treating vocal leukoplakia but also superior voice outcomes. In addition, there were no significant predictive factors related to disease recurrence or progression in our study. Although further large-scale studies are required to validate our results regarding angiolytic lasers, we confirmed that an angiolytic laser is a valid treatment option for vocal fold leukoplakia.
